# Four oral iron supplements for treating iron-deficiency anemia during pregnancy in China: a cost-effectiveness and budget analysis

**DOI:** 10.3389/fpubh.2025.1596874

**Published:** 2025-10-02

**Authors:** Ling Zhang, Zixing Zeng, Biyang Zhang, Hai Gu

**Affiliations:** Center for Health Policy and Management Studies, School of Government, Nanjing University, Nanjing, China

**Keywords:** iron-deficiency anemia, pregnancy, oral iron supplements, cost-effectiveness analysis, budget

## Abstract

**Objectives:**

This study has two primary objectives: (a) to conduct a comparative cost-effectiveness analysis of four commonly used oral iron supplements for treating iron-deficiency anemia during pregnancy in China, including ferrous succinate sustained-release tablets, polysaccharide-iron complex capsules, iron protein succinylate oral solution, and iron dextran oral solution; and (b) to assess the budget impact of including ferrous succinate sustained-release tablets in the National Reimbursement Drug List (NRDL) on national medical insurance expenditures.

**Methods:**

A decision tree model was developed to analyze the cost-effectiveness based on treatment efficacy derived from a network meta-analysis. A sensitivity analysis was conducted to address uncertainties in the parameters. Subsequently, a budget impact analysis model was utilized to calculate the effect of including ferrous succinate sustained-release tablets in the NRDL on the expenditures of employee medical insurance funds, resident medical insurance funds, and the total medical insurance fund expenditures.

**Results:**

The cost-effectiveness analysis showed that ferrous succinate sustained-release tablets are a cost-effective treatment option. When compared to polysaccharide-iron complex capsules, the additional cost per effect of the ferrous succinate sustained-release tablets is $3.23. If these tablets are included in the NRDL, the total medical insurance expenditure on oral iron preparations for treating iron-deficiency anemia in pregnant women is expected to decrease from $160.14 million to $156.82 million between 2025 and 2027.

**Conclusion:**

Ferrous succinate sustained-release tablets are a cost-effective treatment option for iron-deficiency anemia during pregnancy in China.

## 1 Introduction

Anemia is the third leading cause of disability worldwide, accounting for 5.7% of all years of life lost due to disability, making it a significant contributor to the global burden of disease (1). The most common type of anemia during pregnancy is iron-deficiency anemia (IDA), which occurs when there is insufficient iron intake, increased demand, or excessive loss of iron. IDA accounts for about 60% of all anemia cases during pregnancy (2, 3). The primary causes of IDA in pregnant women include increased iron demand (4), inadequate daily iron intake, and poor iron absorption (5–8). In China, 13.9% of women are affected by iron deficiency anemia during pregnancy (9).

As pregnancy progresses, there is an increase in both blood volume and the number of red blood cells to support the growth of the fetus and placental tissues, requiring an additional 600 to 800 mg of iron (10). However, since dietary iron absorption is only about 10% per day, the total amount of iron absorbed from food is relatively low. When iron intake is insufficient, expectant mothers easily develop IDA, which can negatively impact fetal development, maternal health, and overall pregnancy outcomes (11–13). Women with IDA may experience symptoms such as palpitations, shortness of breath, and irritability, and in severe cases, IDA can lead to heart failure and premature birth (14–16). Once a mother’s iron stores are depleted, meeting her iron needs through diet alone becomes challenging (17), making iron supplementation essential.

The treatment of IDA during pregnancy aims to replenish iron stores and restore normal hemoglobin levels (18, 19). Current treatment options include oral iron, intravenous iron, and combined nutritional interventions (20, 21). Oral iron supplementation is an effective, relatively inexpensive, and safe method to treat IDA (22–25). Ferrous salts are preferred due to their superior absorption and bioavailability compared to ferric salts (26, 27). Multivitamin formulas often contain insufficient iron to correct anemia and may include other minerals that hinder iron absorption (28).

Existing economic evaluations mainly compare oral and intravenous iron treatments, with few studies focusing on the different types of oral iron preparations. No definitive conclusions have been reached regarding their economic advantages (29–31). Given the limited medical resources and rising healthcare costs, it is essential to base clinical practices on economic value. Assessing the clinical efficacy and economic value of IDA therapies during pregnancy is crucial for selecting the most appropriate treatment options. In China’s oral iron market, the leading products are polysaccharide iron complex and iron protein succinylate oral solution, while ferrous succinate sustained-release tablets and iron dextran oral solution have smaller market shares (32). Currently, there are no studies comparing their economic feasibility.

To optimize the allocation of medical resources, it is essential to evaluate the economic feasibility of various treatments and conduct a budget impact analysis for medical insurance. This study first assesses the cost-effectiveness of four common oral iron supplements used to treat iron deficiency anemia during pregnancy, focusing on the perspective of the Chinese healthcare system. It then performs a budget impact analysis from the viewpoint of Chinese payers, examining the potential impact of incorporating these supplements into the NRDL on medical insurance expenditures over the next 3 years. This analysis aims to provide decision support and empirical reference for medical insurance policies.

## 2 Methods

### 2.1 Network meta-analysis

We carried out a comprehensive literature search using several databases, including PubMed,^1^ Embase,^2^ the Cochrane Library,^3^ China National Knowledge Infrastructure (CNKI, https://www.cnki.net/), Wanfang Data,^4^ and the VIP Database for Chinese Technical Periodicals (VIP, http://www.cqvip.com/), to identify relevant publications. This search was limited to manuscripts published before March 4, 2024. The detailed search strategy for each database is provided in Supplementary Figure S1. Following predefined inclusion and exclusion criteria, eligible studies were selected for data extraction and analysis using Stata 18.0. The primary efficacy outcome was the total effective rate, defined as the percentage of patients achieving either a markedly effective response (Hb > 110 g/L) or an effective response (an increase in Hb of >20 g/L from baseline). The resulting efficacy estimates were used as inputs for the cost-effectiveness model.

### 2.2 Cost-effectiveness analysis

Cost-Effectiveness Analysis (CEA) is a pharmacoeconomic method used when the same clinical outcome metrics are used as health outputs (33). In this study, the total effective rate was used as the sole outcome indicator for effectiveness, and the incremental cost-effectiveness ratio (ICER) represents the additional cost required to achieve a 1% increase in total effective rate. While no established willingness-to-pay (WTP) threshold exists specifically for this outcome measure in China, we assumed 1 times the 2023 per capita disposable income ($5,523 per 1% increase in total effective rate) as a contextual reference point rather than a strict decision threshold (34–37). Given the uncertainty surrounding appropriate threshold values, our primary cost-effectiveness conclusions are based on probabilistic sensitivity analysis using cost-effectiveness acceptability curves (CEAC), which demonstrate the probability that each intervention represents the most cost-effective option across a continuous range of possible WTP values, providing decision-makers with comprehensive information under parameter uncertainty. The formula for calculating ICER is as follows (38):


$$\begin{array}{l} \text{ICER}=(\text{Cost of Intervention}-\text{Cost of Comparator})\\ /(\text{Outcome of Intervention}-\text{Outcome of Comparator}) \end{array}$$

**Figure 1:**
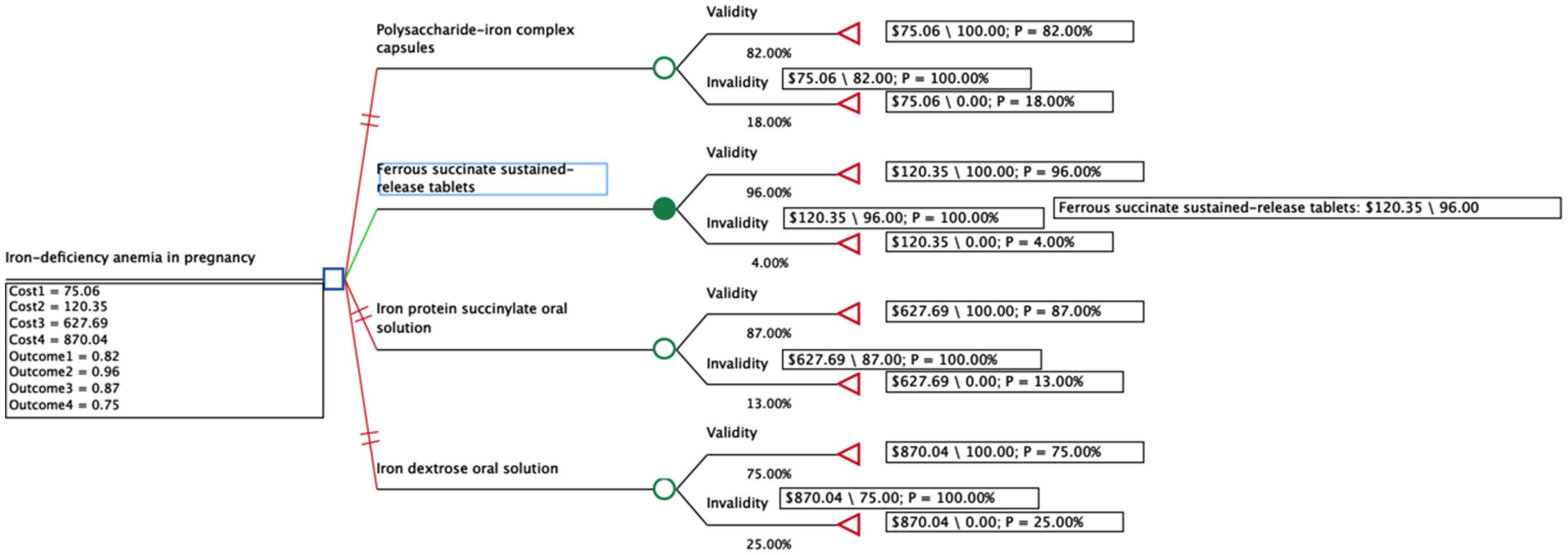
Decision tree model of four common oral iron supplements for the treatment of iron deficiency anemia in pregnancy.

A decision tree model was constructed using TreeAge software to systematically evaluate and compare the cost-effectiveness of four oral iron supplements for treating IDA during pregnancy (39). This modeling approach provides a structured analytical framework to represent the treatment pathway and synthesize evidence on costs and effects, as depicted in Figure 1. The model integrated key parameters, including the clinical effectiveness for each treatment and the direct drug acquisition costs, in order to calculate the primary model outputs. These outputs were the total cost and total effectiveness for each strategy, which were then used to determine the final ICERs. The model covered a 28-week time span, reflecting the standard treatment period where iron supplements are initiated in mid-pregnancy and continued for 3–6 months or until 3 months postpartum (40). From the perspective of the Chinese health system, we used the total effective rate from the network meta-analysis as the health output. Since all medicines are administered orally with low incidence of adverse drug reactions, we assumed high patient compliance and calculated only direct medical costs. Costs were calculated as follows:


$$\begin{array}{l} \text{Cost}(C)=\text{average daily treatment cost}\times \text{treatment}\\ \text{period}=\text{unit drug cost}\times \text{dose}\times \text{number of times}\\ \mathrm{per}\;\mathrm{day}\times \text{number of days of treatment} \end{array}$$

### 2.3 Sensitivity analysis

To evaluate the robustness of model results, we performed one-way deterministic sensitivity analyses by individually varying each parameter listed in Table 1. For effectiveness parameters (total effective rates), we used the 95% credible intervals (CI) derived from our Bayesian network meta-analysis. For cost parameters, we varied all cost variables by ±10% from the base case values to account for potential price fluctuations and regional cost variations.

**Table 1:** Sensitivity analysis parameter settings.

Parameters	Base value	Minimum value	Maximum value	Probability distribution
Total cost/($)				
Polysaccharide-iron Complex Capsules (Cost 1)	75.06	67.55	82.57	gamma
Ferrous Succinate Sustained-release Tablets (Cost 2)	120.35	108.32	132.39	gamma
Iron Protein Succinylate Oral Solution (Cost 3)	627.69	564.92	690.46	gamma
Iron Dextrose Oral Solution (Cost 4)	870.04	783.04	957.04	gamma
Outcome^a^/(%)				
Polysaccharide-iron Complex Capsules (Outcome 1)	82.00	72.00	91.00	beta
Ferrous Succinate Sustained-release Tablets (Outcome 2)	96.00	87.00	99.00	beta
Iron Protein Succinylate Oral Solution (Outcome 3)	87.00	65.00	100.00	beta
Iron Dextrose Oral Solution (Outcome 4)	75.00	64.00	84.00	beta

a Outcome refers to the total effective rate (%) for each oral iron supplement, representing the percentage of patients who achieved successful treatment response as derived from the network meta-analysis.

A probabilistic sensitivity analysis was performed to evaluate overall parameter uncertainty. In this analysis, parameters for cost and effectiveness were simultaneously sampled from their respective probability distributions (Gamma for costs; Beta for effectiveness) over 1,000 Monte Carlo simulations. The results of these simulations were used to generate a cost-effectiveness acceptability curve, which illustrates the probability of each intervention being the most cost-effective option across a range of WTP thresholds.

### 2.4 Budget impact analysis

A budget impact analysis (BIA) model estimated expected expenditure changes for the Chinese healthcare system (41), comparing two scenarios: ferrous succinate sustained-release tablets retained in or removed from the National Reimbursement Drug List (NRDL).

We used the 2020 population size of China as a baseline and combined it with data from the National Statistical Bulletin on the Development of Basic Medical Security. This data shows that the medical insurance participation coverage remains stable at over 95%. Therefore, we assumed a participation rate of 95% for this analysis (Table 2).

**Table 2:** Target number of patients from 2025 to 2027 (10,000 people).

Category	2025	2026	2027
National population	141024.00	140864.00	140669.00
Maternal population	1410.24	1408.64	1406.69
Diseased population	196.02	195.80	195.53
Target population	186.22	186.01	185.75
Employee population	51.77	51.71	51.64
Urban and rural residents	135.38	135.23	135.04

The market share projection is mainly based on the sales data of 2023 in the CHPA released by IQVIA, combined with the market share change data provided by enterprises from 2021 to 2023 and the results of expert interviews for prediction. We assume that the average annual growth rate of the market share of ferrous succinate sustained-release tablets is 1% when they are retained in the NRDL, and 0.7% when they are removed from the NRDL (Table 3).

**Table 3:** Percentage of oral iron drug utilization for each of the two scenarios, 2025 to 2027 (%).

Scenario	Name of drug	2023	2025	2026	2027
Scenario 1	Ferrous Succinate Sustained-release Tablets	20.2	22.0	22.9	23.8
	Polysaccharide-iron Complex Capsules	33.0	36.6	38.4	40.2
	Iron Protein Succinylate Oral Solution	31.2	29.8	29.1	28.4
	Iron Dextrose Oral Solution	8.8	7.8	7.3	6.8
Scenario 2	Ferrous Succinate Sustained-release Tablets	20.2	22.2	23.2	24.2
	Polysaccharide-iron Complex Capsules	33.0	36.4	38.1	39.8
	Iron Protein Succinylate Oral Solution	31.2	29.4	28.5	27.6
	Iron Dextrose Oral Solution	8.8	7.2	6.6	6.0

The budget impact analysis employed a 3-year time horizon (2025–2027). From the Chinese health insurance payer perspective, the analysis examined outpatient expenses for IDA during pregnancy, including only medication and routine examination costs covered by reimbursement. Based on National Healthcare Security Administration guidelines, we assumed 80% reimbursement rates for Class B drugs, with specific rates of 70% for urban workers and 50% for urban and rural residents.

## 3 Results

### 3.1 Network meta-analysis

The network meta-analysis included 14 different therapeutic interventions from 28 studies encompassing 3,692 patients, forming a well-connected evidence network (Supplementary Figure S2). Risk of bias assessment showed acceptable methodological quality across included studies (Supplementary Figure S3). Using ferrous succinate sustained-release tablets as reference, the network meta-analysis generated effectiveness estimates for all interventions (Supplementary Figure S4). From this comprehensive network, we derived the following total effective rates for our four target interventions: polysaccharide-iron complex capsules (82.0%), ferrous succinate sustained-release tablets (96.0%), iron protein succinylate oral solution (87.0%), and iron dextran oral solution (75.0%), which served as efficacy parameters in our cost-effectiveness model.

### 3.2 Cost-effectiveness analysis

#### 3.2.1 Base-case analysis

In this study (Table 4), ferrous succinate sustained-release tablets had the lowest ICER at $3.23, meaning the incremental cost per 1% increase in total effective rate was $3.23. This figure is significantly lower than the patient’s WTP threshold of $8,285, making ferrous succinate sustained-release tablets a favorable treatment option. On the other hand, Iron protein succinylate oral solution had a very high ICER of $110.53, while iron dextran oral solution was a dominated strategy, being both more costly and less effective than the comparator.

**Table 4:** Results of the base-case analysis.

Regimen	Outcome/(%)	Total Cost/($)	Incremental outcome/(%)	Incremental cost/($)	Average CER/($/%)	ICER/($/%)
Polysaccharide-iron Complex Capsules	82.00	75.06	0	0	0.85	—
Ferrous Succinate Sustained-release Tablets	96.00	120.35	14.00	45.29	1.25	3.23
Iron Protein Succinylate Oral Solution	87.00	627.69	5.00	552.63	7.08	110.53
Iron Dextrose Oral Solution	75.00	870.04	−7.00	794.98	11.63	Dominated

#### 3.2.2 Sensitivity analysis

**Figure 2:**
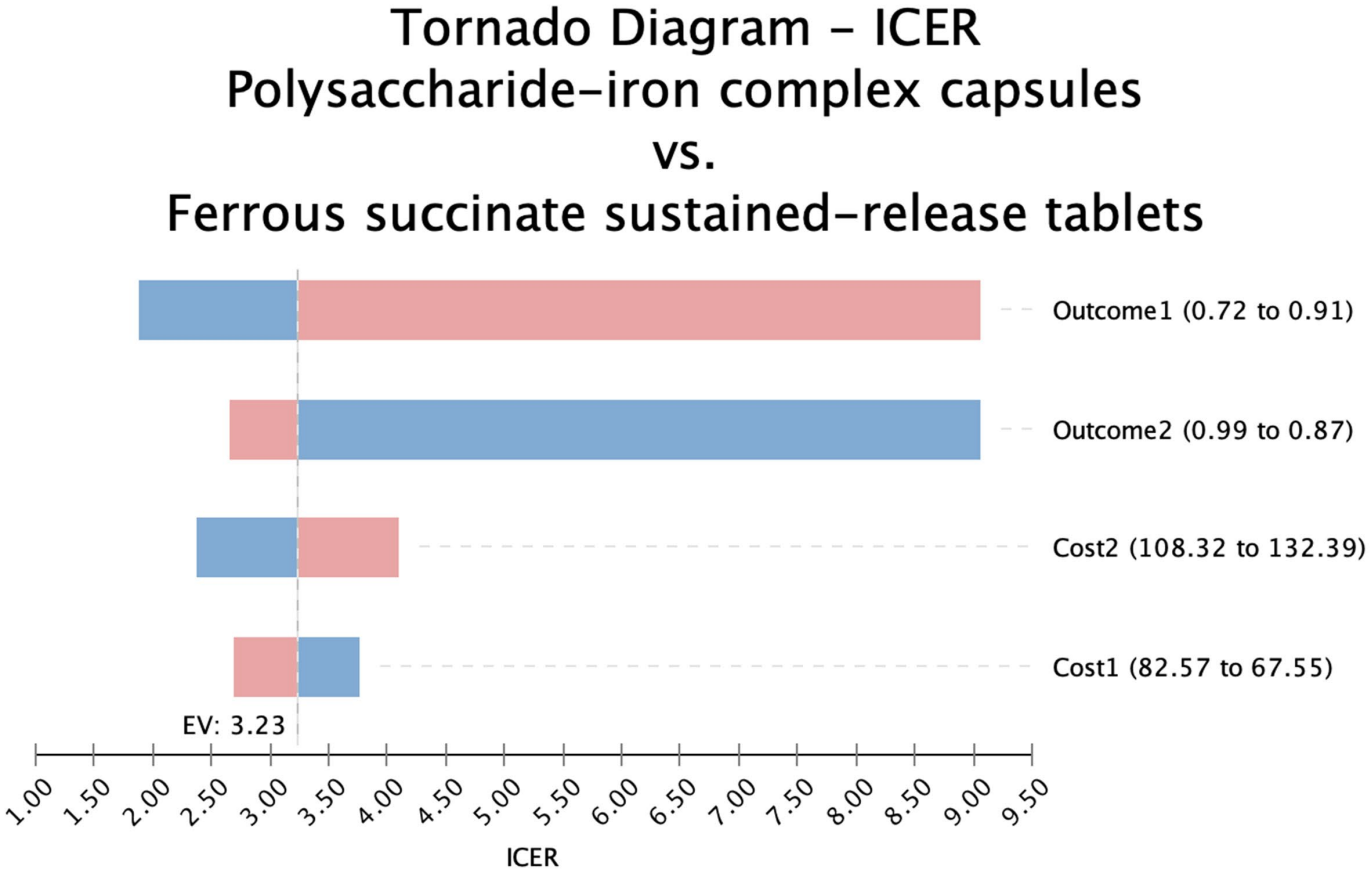
Tornado diagram-polysaccharide iron complex vs ferrous succinate sustained release tablets.

The one-way sensitivity analysis confirmed that these two interventions were not favorable options. The ICER for iron protein succinylate oral solution remained significantly higher than the WTP threshold across the tested parameter ranges, while iron dextran oral solution consistently remained a dominated strategy (Supplementary Figures S5 and S6). In contrast, ferrous succinate sustained-release tablets showed ICER values consistently lower than the WTP threshold, confirming its cost-effectiveness advantage (Figure 2).

**Figure 3:**
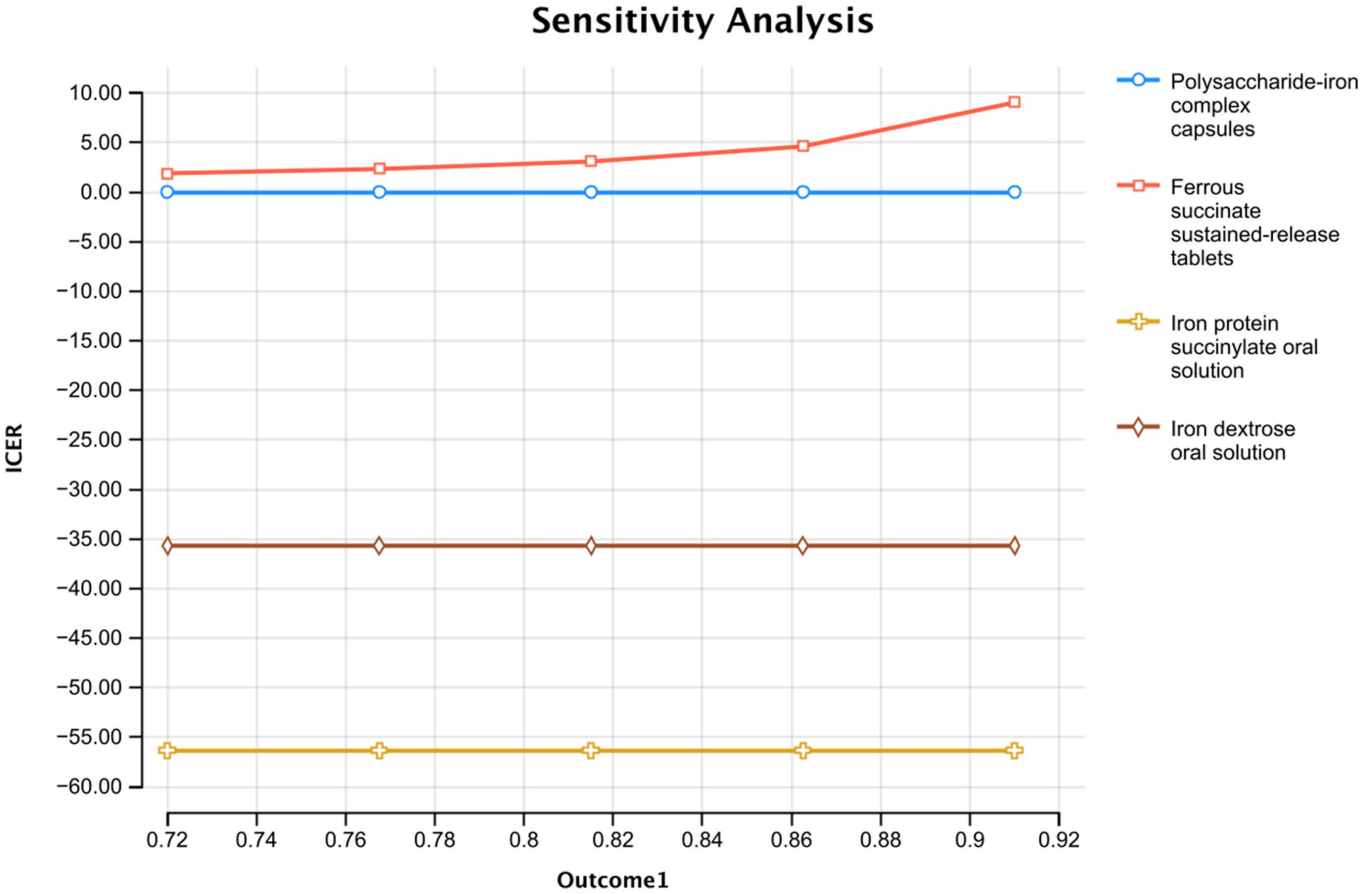
Single factor sensitivity analysis of the effective rate of polysaccharide iron capsule treatment.

The effectiveness of polysaccharide-iron complex capsules significantly influenced the outcomes, ranging from 87.0 to 99.0% within a 95% credible interval according to the network meta-analysis. The ICER comparisons demonstrated that both Iron protein succinylate oral solution and Iron dextran oral solution were consistently less favorable across this range (Figure 3), while ferrous succinate sustained-release tablets remained stable and advantageous across most effectiveness variations.

**Figure 4:**
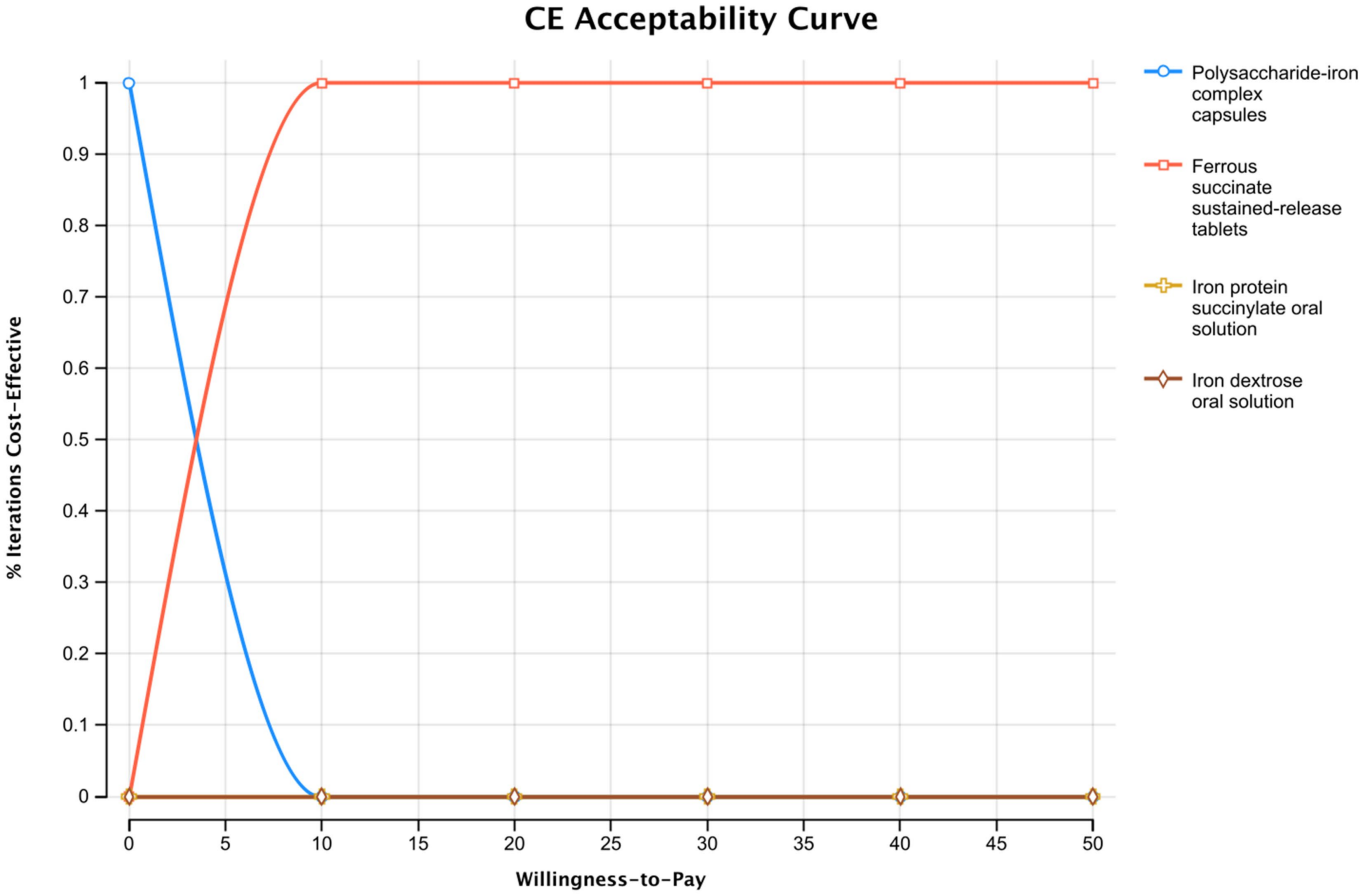
Monte Carlo cost-effectiveness acceptability curve.

The results of the probabilistic sensitivity analysis are presented in the cost-effectiveness acceptability curve in Figure 4. The curve shows that for any WTP value above the calculated ICER of $3.23 per 1% increase in total effective rate, ferrous succinate sustained-release tablets have the highest probability of being the most cost-effective option. The analysis demonstrates a rapid increase in this probability, reaching nearly 100% at a WTP of approximately $10. This indicates that the economic advantage of ferrous succinate sustained-release tablets is robust and confirmed at a very low WTP threshold, and it remains the definitive optimal choice across all higher values, including the contextual reference point of $5,523. These probabilistic results strongly support the base-case findings under parameter uncertainty.

### 3.3 Budget impact analysis

Under the scenario where ferrous succinate sustained-release tablets are withdrawn from the NRDL, the total medical insurance payment for oral iron supplementation in pregnant patients with IDA is projected to increase from approximately $149.70 million to $152.91 million from 2025 to 2027. The total Medicare payment for insured employees increases from about $52.20 million to $53.32 million, and for insured residents, it increases from around $97.50 million to $99.59 million (Table 5).

**Table 5:** Changes in total Medicare fund payments from 2025 to 2027 under the withdrawal scenario (Millions of Dollars).

Year	Partial payment for insured employees	Partial payment for insured residents	Total Medicare payments
2025	52.20	97.50	149.70
2026	52.76	98.56	151.32
2027	53.32	99.59	152.91

Conversely, if ferrous succinate sustained-release tablets remain in the NRDL, the total Medicare payment for treating pregnant patients with iron-deficiency anemia with oral iron supplements from 2025 to 2027 is projected to decrease from approximately $160.14 million to $156.82 million. The total Medicare payment for insured employees decreases from about $55.84 million to $54.68 million, and for insured residents, it decreases from around $104.30 million to $102.14 million (Table 6).

**Table 6:** Changes in total Medicare fund payments from 2025 to 2027 under the access scenario (Millions of Dollars).

Year	Partial payment for insured employees	Partial payment for insured residents	Total Medicare payments
2025	55.84	104.30	160.14
2026	55.27	103.23	158.50
2027	54.68	102.14	156.82

## 4 Discussion

This study evaluated the pharmacoeconomic value of four common oral iron supplements for treating IDA in pregnancy within the Chinese healthcare system. Our findings revealed that ferrous succinate sustained-release tablets offer a highly favorable cost-effectiveness profile, with a low incremental cost-effectiveness rate ($3.23) falling significantly below the willingness-to-pay threshold. This conclusion’s robustness was confirmed through consistent results across the base-case, one-way, and probabilistic sensitivity analyses, highlighting their potential as a priority treatment option. In contrast, the other evaluated alternatives were found to be economically unfavorable.

Our cost-effectiveness analysis has several important limitations. First, the cost analysis focused solely on direct drug acquisition costs and excluded expenses related to laboratory tests, registration fees, and medical consultations. Second, we excluded indirect costs such as productivity losses, transportation costs, and caregiver burden, which could be substantial from a societal perspective. Third, our analysis assumed perfect patient adherence based on controlled clinical trial data, while real-world adherence rates may vary significantly between different iron formulations due to dosing frequency, side effect profiles, and patient preferences. Finally, our effectiveness data derived from clinical trials may not fully represent diverse patient populations in routine practice. While these limitations may introduce some bias, the relative cost-effectiveness comparisons remain valid as most excluded costs would be consistent across treatment options. Therefore, the results still provide valuable insights for policy decisions.

An important consideration concerns the selection of cost-effectiveness thresholds. In the absence of established thresholds specific to iron deficiency anemia treatment during pregnancy in China, we referenced per capita disposable income for threshold estimation. However, individual willingness-to-pay varies considerably across different stakeholders. Our cost-effectiveness acceptability curve analysis addresses this uncertainty by demonstrating the probability of cost-effectiveness across varying threshold values, enabling healthcare providers, pregnant women, and health system decision-makers to apply criteria appropriate to their specific circumstances and resource constraints.

Using the budget impact analysis model, we evaluated how the inclusion of ferrous succinate sustained-release tablets in the NRDL affects payments to the employee health insurance fund, the resident health insurance fund, and the overall expenditure of the health insurance fund. When ferrous succinate sustained-release tablets are included for the treatment of IDA, the total payment from the health insurance fund decreases. Between 2025 and 2027, the net impact on health insurance fund payments is projected to decline from approximately $9.65 million to $3.13 million. Specifically, the incremental expenditure for employee health insurance will fall from about $3.37 million to $1.09 million, while the incremental expenditure for resident health insurance will decrease from approximately $6.29 million to $2.04 million.

As the treatment of IDA during pregnancy is the main application scenario for ferrous succinate sustained-release tablets, and the main sales of oral iron products such as ferrous succinate sustained-release tablets are derived from this therapeutic area, this study limits the target population to patients with IDA during pregnancy and does not account for other uses of ferrous succinate sustained-release tablets. Therefore, the estimation of the impact on the medical insurance fund may be biased. Additionally, the study assumed that the health insurance payment ratio remained unchanged and did not consider the actual higher payment ratios. It only considered routine outpatient treatment and not hospitalization of patients with IDA during pregnancy, which may introduce bias in the measurement of the health insurance fund payment. Finally, the sales data used in the study are speculative based on historical data and expert interviews, without fully considering policy factors such as bulk purchasing, reform of health insurance payment methods, and the impact of possible low-priced generics in the same therapeutic area, which may lead to biased market share estimation.

## 5 Conclusion

Based on our comprehensive economic evaluation, ferrous succinate sustained-release tablets represent the most cost-effective option for treating iron deficiency anemia during pregnancy in China, with an incremental cost-effectiveness ratio of $3.23 per 1% increase in treatment effectiveness. Inclusion of ferrous succinate sustained-release tablets in the National Reimbursement Drug List would reduce national medical insurance expenditures year by year from 2025 to 2027 while ensuring optimal therapeutic outcomes. These findings provide strong economic evidence to support healthcare policy decisions favoring ferrous succinate sustained-release tablets as a priority treatment option for iron deficiency anemia in pregnancy.

## Data Availability

The original contributions presented in the study are included in the article/Supplementary material, further inquiries can be directed to the corresponding author.

## Footnotes

^1^ https://pubmed.ncbi.nlm.nih.gov/

^2^ https://www.embase.com/

^3^ https://www.cochranelibrary.com/

^4^ https://www.wanfangdata.com.cn/
