# Supplementary material for: Four oral iron supplements for treating iron-deficiency anemia during pregnancy in China: a cost-effectiveness and budget analysis
Source: Front Public Health. 2025 Oct 2;13:1596874. doi: 10.3389/fpubh.2025.1596874 (PMC12528109; doi:10.3389/fpubh.2025.1596874)
Supplement: Supplementary file 1 [file Data_Sheet_1.docx]

Supplementary Material

# 1. Supplementary Figure S1.


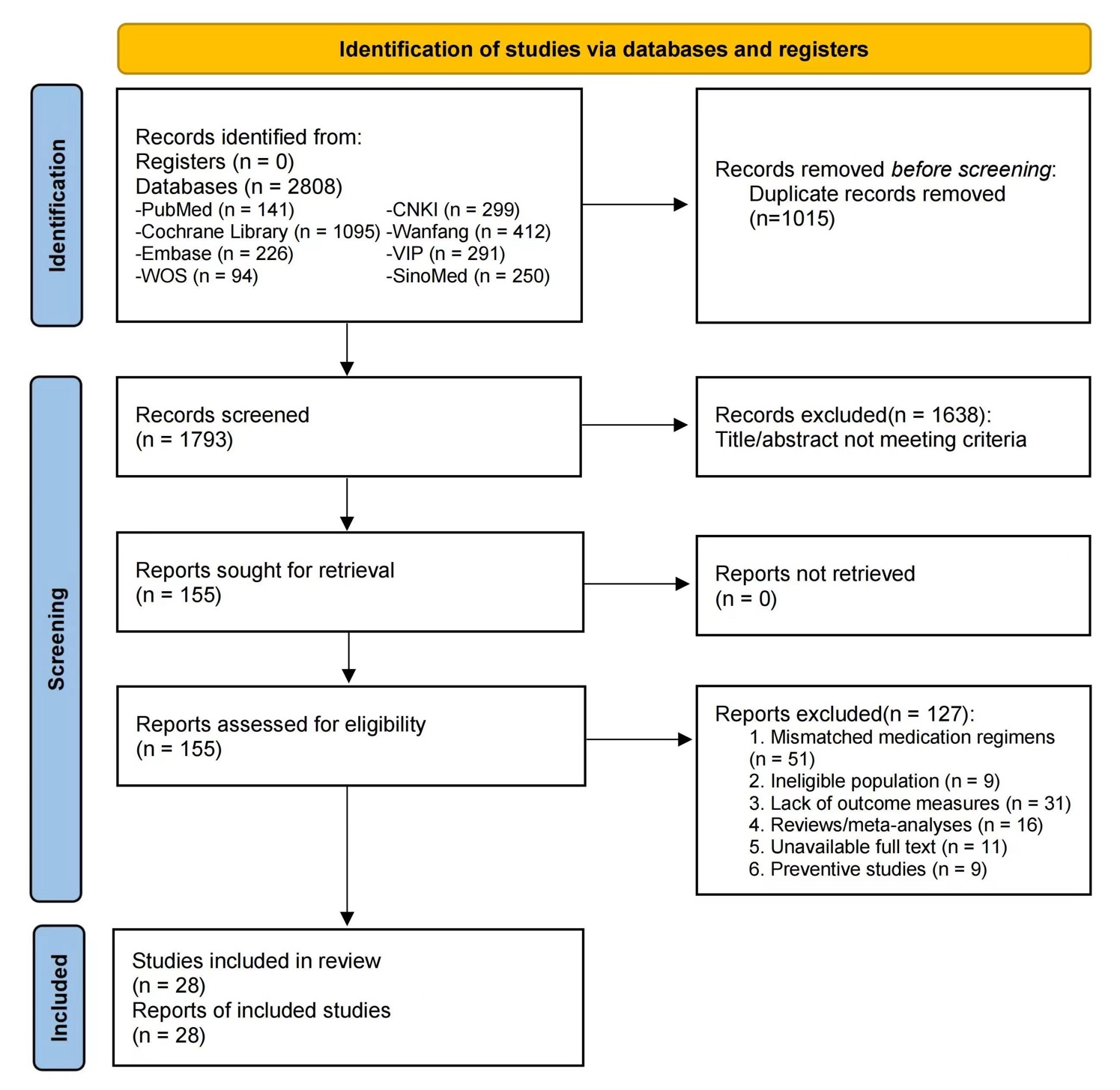


**2. Supplementary** **Figure S2. Network evidence plot for total effective rate**

**
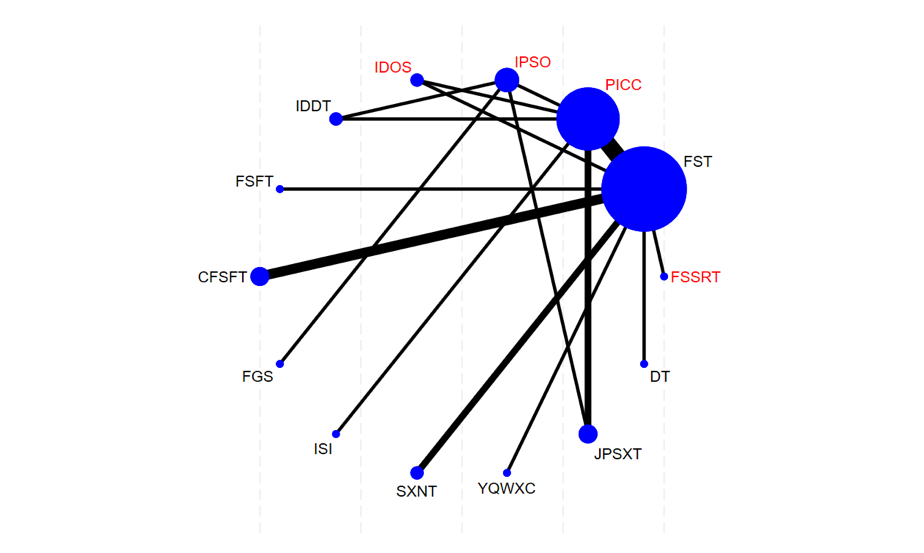
**

**3. Supplementary** **Figure S3. Risk of bias assessment of included studies**

**
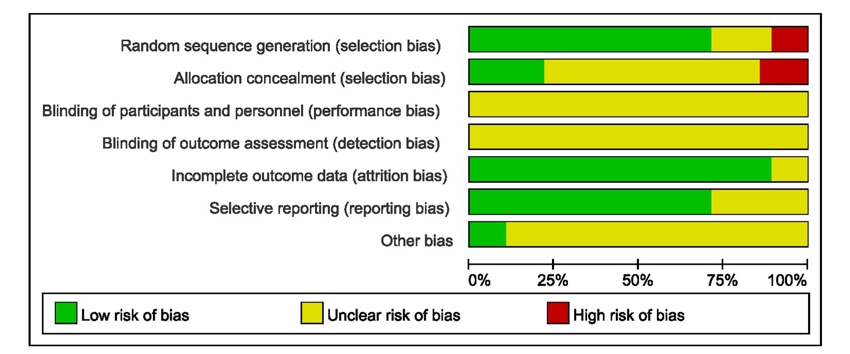
**

**4. Supplementary** **Figure S4. Forest plot for total effective rate**

**
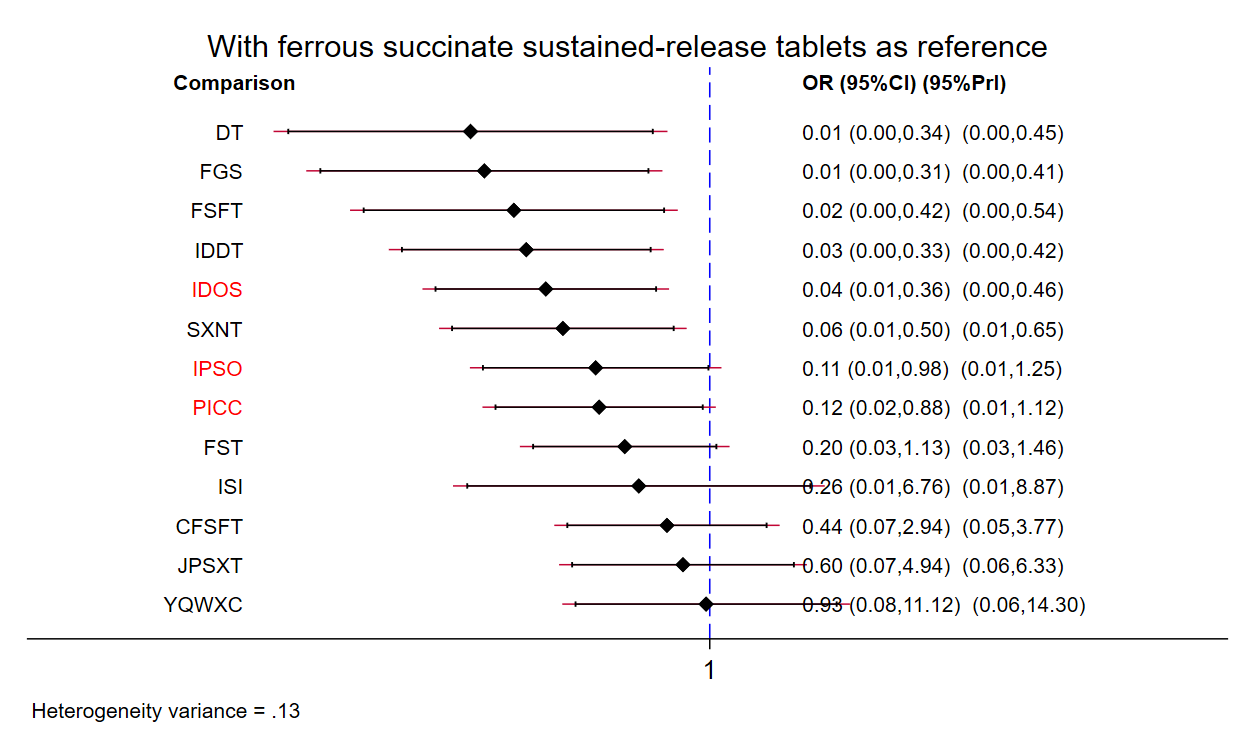
**

(Abbreviations used in the figures are as follows: FSSRT, Ferrous succinate sustained-release tablets; FST, Ferrous succinate tablets; PICC, Polysaccharide-iron complex capsules; IPSO, Iron protein succinylate oral solution; IDOS, Iron dextran oral solution; IDDT, Iron dextran dispersible tablets; FSFT, Ferrous sulfate tablets; CFSFT, Compound ferrous sulfate and folic acid tablets; FGS, Ferrous gluconate syrup; ISI, Iron sucrose injection; SXNT, Shengxuening tablets; JPSXT, Jianpishengxue tablets; YQWXC, Yiqiweixue capsules; DT, Dietotherapy.)

**5. Supplementary** **Figure S5. Tornado Diagram-Polysaccharide Iron Complex VS polysaccharide iron complex capsules**

**
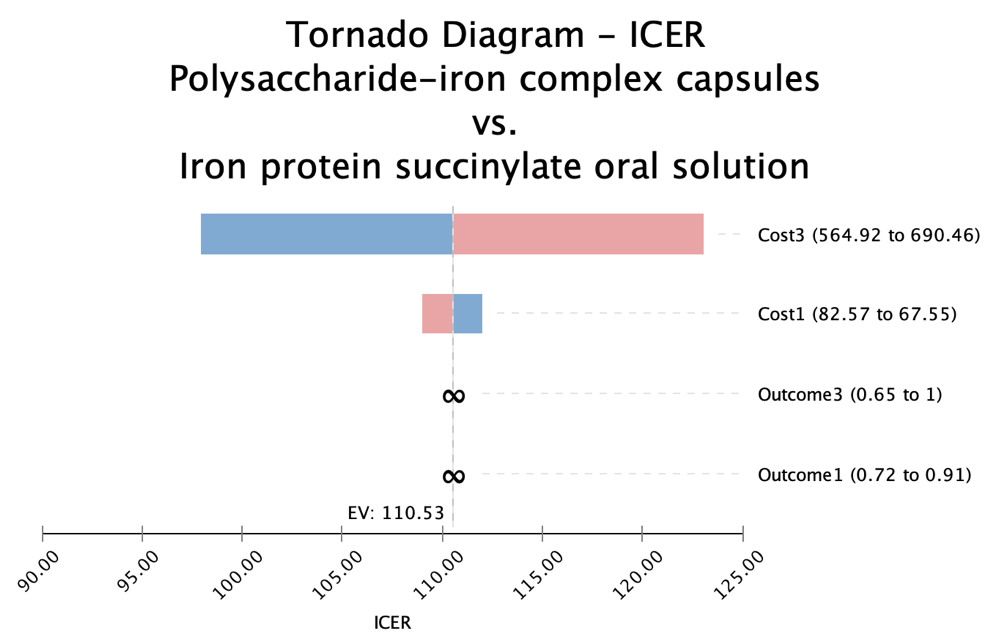
**

**6. Supplementary** **Figure S6. Tornado Diagram-Polysaccharide Iron Complex VS protein succinate iron oral solution**

**
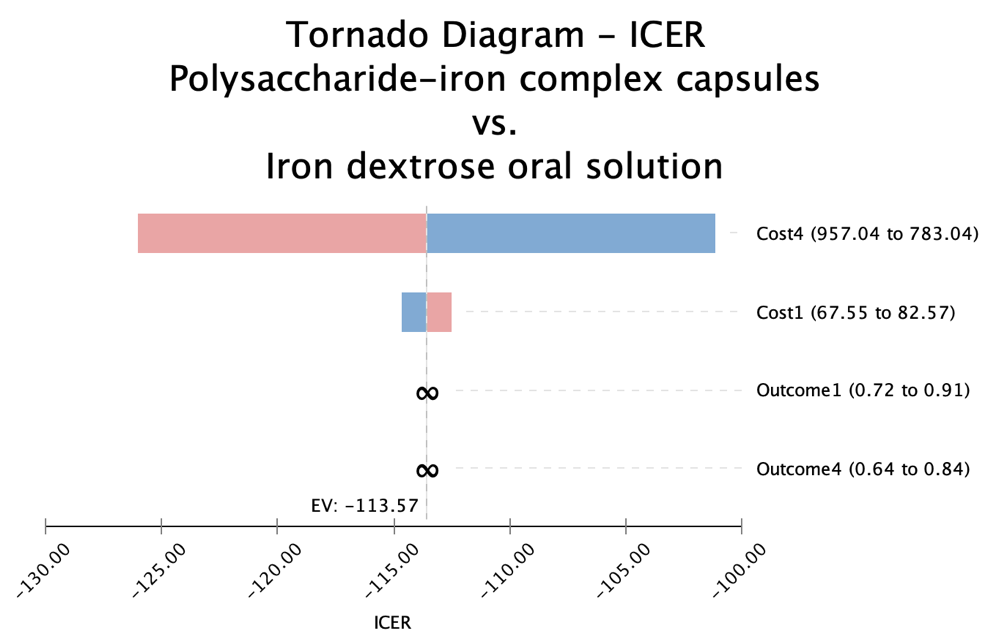
**
